# Structure–Property Associations: Breaking Paradigms for Linking Chemical Structures and Biological Properties in Drug Discovery

**DOI:** 10.1002/cmdc.202500847

**Published:** 2025-11-25

**Authors:** Edgar López‐López, J. Israel Espinoza‐Castañeda, Karina Martinez‐Mayorga, José L. Medina‐Franco

**Affiliations:** ^1^ DIFACQUIM Research Group School of Chemistry Department of Pharmacy National Autonomous University of Mexico Mexico City 04510 Mexico; ^2^ Institute of Chemistry Campus Merida National Autonomous University of Mexico Merida‐Tetis Highway, Km. 4.5 Ucu 97357 Yucatan Mexico; ^3^ Department of Chemistry and Graduate Program in Pharmacology Center for Research and Advanced Studies of the National Polytechnic Institute Mexico City 07360 Mexico

**Keywords:** bioactivity disruption, chemoinformatics, computer‐aided molecular design, drug design, structure–activity relationships

## Abstract

Structure–activity relationships (SARs) are a cornerstone of drug discovery, aiming to elucidate the connection between chemical structures and their biological properties. While widely applied in medicinal chemistry and computer‐aided molecular design, SAR traditionally assumes a direct connection (i.e., relations) between chemical structures and their activity. However, given the complexity of biological responses, these connections are often better described as associations rather than strict relationships. Beyond semantics, relations usually imply a deterministic or functional mapping, whereas, associations are treated with statistical tools that capture probabilistic patterns without assuming causality. Adopting an association‐based perspective helps to avoid overstated claims and manage uncertainty more realistically. In this article, structure–property associations (SPAs) are proposed as a more accurate framework to capture the connection between chemical structures and their properties in the context of drug discovery. SPA is particularly emphasized in describing the associations between chemical structures and biological activity across different experimental levels, including both in vitro and in vivo assays.

## Graphical abstract

The figure contrasts traditional structure–activity relationships, which assume direct links between chemical structures and biological activity, with structure–property associations (SPAs), which capture indirect connections across multiple biological scales. SPAs provide a more realistic framework to understand complex biological responses in drug discovery.

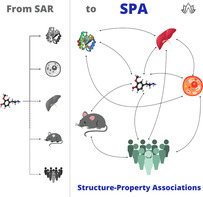

## 1 Introduction

Exploring structure–activity relationships (SARs), and more broadly, structure–property relationships (SPRs) remains a cornerstone of medicinal chemistry. These analyses provide essential insights for guiding the rational design and optimization of molecules by revealing how specific structural features influence biological activity or other relevant properties.

While molecular structure plays a central role in such analysis, it is essential to recognize that a compound's behavior is also shaped by its surrounding “environment”—including factors such as concentration, for example, compound dose, pH, and oxygen levels of the system; compound formulation; and biological context such as receptor conformational dynamics.^[^ ^1^ ^]^ This complexity underscores the need for caution when interpreting SARs in isolated systems. Understanding how structure interacts with dynamic biological systems is crucial for making reliable predictions and advancing effective drug discovery strategies.

In the field of computer‐aided drug design (CADD), or more broadly computer‐aided bioactive compound design (CABioCD), SAR serves as the foundation for many computational approaches. For example, the seminal work of Hansch–Fujita was aimed at establishing a qualitative assessment of the SAR—QSAR.^[^ ^2^ ^,^ ^3^ ^]^ Early QSAR studies were done with analog series and relatively low variability relationships.^[^ ^3^ ^,^ ^4^ ^]^ However, fundamental concepts in chemistry, such as additivity and transferability,^[^ ^1^ ^]^ do not hold in biological systems. A case in point of the transferability is the notion of functional groups. Importantly, the high complexity of biological systems challenges such generalizations.^[^ ^1^ ^]^ For decades, proper and rigorous validation of QSAR models has been debatable.^[^ ^5^ ^,^ ^6^ ^]^ QSAR has been evolving and has been integrated into modern machine‐learning methods that play a central role in artificial intelligence (AI)‐driven drug discovery.

The identification of “activity cliffs”,^[^ ^7^ ^]^ whose presence can be due to artifacts such as errors in biological activity measurements,^[^ ^8^ ^]^ and recently framed as “bioactivity disruptors”,^[^ ^1^ ^]^ introduces additional complexity to the connection between chemical structures and their biological response. Indeed, it has been recently argued that, alternatively called “structural activity cliffs,” point specifically to modifications in activity due to changes caused strictly by modifications in chemical structures.^[^ ^1^ ^]^ In this context, bioactivity disruption has been defined recently as an “abrupt or nonlinear interference in bioactivity arising from concentration‐dependent or dynamic biological processes, rather than structural changes alone“.^[^ ^1^ ^]^ Furthermore, for complex systems, where several variables are involved, it is more appropriate to assess associations rather than relationships. The type (interrelated and complex) and amount of variables need to be treated statistically, via associations, rather than to establish a direct dependence (relations) between the descriptors and the biological activity.

In practice, many studies use simplified or idealized systems, consistent with the principle of reductionism,^[^ ^9^ ^]^ conventionally related to physicochemical and chemical properties or reduced to a small dataset. Despite this, SAR/SPR terminology is widely used in both the scientific literature and the teaching of medicinal chemistry and CADD‐related courses, which, as discussed herein, may lead to misconceptions and misinterpretations.

Herein, we propose the concept of structure–property association (SPA) as a more appropriate and flexible framework for describing the link between chemical structures and biological effects, particularly within complex biological systems. Beyond a mere change in terminology, finding and analyzing associations rather than relationships at both qualitative and quantitative levels, may significantly impact how we interpret and statistically assess the connection between chemical structures and their properties, with emphasis on biological effects or on clinical outcomes.

## 2 The Basis of Associations and Relationships

According to the Oxford English Dictionary, a relationship is defined as ‘the way in which two or more people or things are connected, or the state of being connected’, often implying a structured or functional link that may suggest causality or dependency. In contrast, an association is described as ‘a connection or cooperative link between people or organizations’ or, more broadly, as ‘a connection between ideas or things’. Although the terms “relationship” and “association” are sometimes used interchangeably to describe a form of connection, they carry distinct methodological implications in the context of data analysis.^[^ ^10^ ^]^ In mathematics,^[^ ^11^ ^]^ a relationship often implies a structured, potentially causal connection, while an association typically refers to a noncausal co‐occurrence between variables without necessarily implying causation or directionality, a very general connection where one variable provides information about another. This distinction is critical for the accurate interpretation of data‐driven models, as understanding the connection between variables has significant methodological and interpretive implications.

### 2.1 Exploratory Data Analysis (EDA)

EDA serves as a critical first step in drug discovery, ensuring the validity and applicability of results by examining chemical datasets for patterns, anomalies, for example, structural activity cliffs (vide supra), and preliminary associations before proceeding to formal modeling of relationships.^[^ ^12^ ^,^ ^13^ ^]^ EDA facilitates a comprehensive understanding of data distribution, trends, and potential biases.

EDA employs a combination of statistical methods and visualizations to identify associations (statistical co‐occurrences) and relationships (predictive dependencies) within chemical data. To identify associations, correlation coefficients, such as Pearson's (for linear associations) and Spearman's (for monotonic, nonlinear associations), are calculated to quantify the strength and direction between variables, such as molecular descriptors and biological activity. For categorical variables, chi‐square test (χ^2^) assesses the statistical significance of associations, with *p*‐values indicating the likelihood of co‐occurrence. Visual tools, including correlation heatmap and pair plots, reveal multivariable and bivariable patterns, respectively. Clustering techniques, such as hierarchical, identify groups of compounds with similar properties, suggesting potential associations.

To probe potential relationships, EDA is also applied to assess the predictive suitability of variables for formal modeling. Scatter plots visualize bivariate relationships, while preliminary regression analysis evaluates the feasibility of functional dependencies, using metrics like R^2^ and root mean square deviation (RMSD) to assess fit. Data distributions are examined via histograms and boxplots to validate assumptions required for models like multiple linear regression or artificial neural networks.

## 3 Examples of Relationships in Drug Discovery

In early stages of drug discovery and development, the notion of relationships has traditionally been framed in relatively direct terms, most often through SAR. These models typically assume that a compound's structural features can be systematically linked to a single biological effect or property, offering a straightforward framework to guide lead optimization or help understand the basis of biological activity, and rationalize properties at the structural level. Such relationships have been instrumental in generating hypotheses, prioritizing chemical libraries, and refining pharmacokinetic or safety profiles during preclinical development.^[^ ^14^ ^]^ However, while these relationships have proven valuable for addressing well‐defined therapeutic targets, they provide only a partial view of the multifaceted interactions that emerge in more complex disease contexts.

**Table** **1** summarizes examples of relationships—qualitative and quantitative—frequently studied in drug discovery projects between structure‐based measurements and the property that is assessed. The list of examples is not exhaustive but is deemed to point out cases where a relationship might be explored and, eventually, quantitatively measured. The table includes the type of experiments or experimental models.

**Table 1:** Examples of structure‐derived calculations and their relationships with experimental data.

Structure‐derived calculation	Measurement (experimental model)
Free binding energy	K_i_ and K_d_ from isothermal calorimetry, binding kinetics
Binding pose; predicted binding mode	RMSD from X‐ray crystal structure; data from NMR of ligand‐target complexes
Tautomer stability	Spectroscopy (e.g., UV–Vis, NMR, and IR)
Redox potentials	Electrochemical measurements (e.g., cyclic voltammetry)
Protonation state	NMR spectroscopy, potentiometric titration, or calorimetry
Low‐energy conformation	X‐ray crystallography
logP and logD predictions	Experimental determination of octanol/water partition and distribution coefficients
Biotransformation predictions	LC‐MS/MS analysis of metabolites generated in liver microsomes

## 4 Examples of Associations in Drug Discovery

In modern drug discovery, the association between a compound's structure and its biological effects is often more complex than what traditional SAR would indicate. This complexity becomes particularly evident in the context of multifactorial and chronic diseases such as Alzheimer's disease, diabetes, or cancer, where a single molecular target is rarely sufficient to drive therapeutic efficacy. Instead, drug candidates often exhibit polypharmacological profiles—interacting with multiple targets simultaneously—which can lead to synergistic or antagonistic biological effects.^[^ ^15^, ^16^, ^17^ ^]^ In this scenario, structure–multiple activity relationships have been proposed to describe how a single chemical scaffold may relate to various biological activities.^[^ ^18^ ^]^ However, the term *structure–multiple activity associations* may offer a more accurate representation, acknowledging that many of these connections are correlative rather than causative.

These associations span multiple layers of biological complexity, from in vitro enzymatic inhibition and receptor modulation to in vivo pharmacokinetic behaviors and clinical outcomes. As illustrated in **Table** **2**, structural‐derived calculations such as docking scores, pharmacophore models, and ADMET (absorption, distribution, metabolism, excretion, and toxicity) predictions are frequently linked to measurements like IC_50_ values, bioavailability, toxicity, or even therapeutic efficacy.^[^ ^18^, ^19^, ^20^ ^]^ Recognizing and leveraging these structures—measurement associations, especially in the context of systemic diseases—can enhance predictive modeling and facilitate rational drug design efforts aimed at modulating complex biological networks rather than isolated targets.

**Table 2:** Examples of structural calculations and their associated biological measurements.

Structurally derived calculation	Measurement
Docking scores; Similarity values	IC_50_, percentage of inhibition or apparent binding affinity
Target predictions	K_i_, IC_50_, target engagement or functional modulation (agonist/antagonist)
Pharmacophore modeling	Selective activity across target families
ADMET predictions	Half‐life (T_1/2_), clearance, bioavailability, volume of distribution, and toxicity
Molecular dynamics (conformational sampling)	Functional efficacy, target stabilization, or ligand internalization
Metabolism prediction	Metabolite formation, biotransformation pathways, or drug–drug interactions
Side effects prediction	Adverse clinical events or off‐target effects
Polypharmacology profiling (multitarget prediction)	Synergistic/antagonistic effects, unintended pharmacology, or network‐level effects
Pathway/network mapping (systems biology)	Clinical outcomes, therapeutic efficacy, or systemic perturbations
Physicochemical property prediction (LogP, PSA, etc.)	Experimental permeability, absorption, or distribution of ligand tautomeric forms
Toxicophore identification	Preclinical toxicity, mutagenicity, or cardiotoxicity risk
Clinical outcome prediction	Therapeutic response, disease progression, or adverse outcomes in specific populations

Despite the remarkable advances in predictive modeling—particularly those harnessing AI methods—that have enabled new strategies in systematic drug candidate screening and optimization, the reality remains sobering: multiple systematic studies have demonstrated that there is no direct, reliable correlation between predicted properties, for example, receptor‐binding affinity, and their experimental values.^[^ ^21^ ^,^ ^22^ ^]^

Spanning drug sensitivity in cell lines, in vivo xenografts, population‐based pharmacokinetics (PK)/pharmacodynamics (PD) models, and causal machine learning in clinical data, it remains clear that these models often capture associations with outcomes rather than delivering precise quantitative projections. For example, Kurilov et al. assessed different modeling strategies using data from cell lines and xenografts for seven drugs. While drug response predictions trained on cell‐line data showed moderate performance (correlation ≈0.5) in an erlotinib‐treated xenograft cohort, they failed for other drugs like gemcitabine or paclitaxel.^[^ ^23^ ^]^ Similarly, Pouryahya et al. developed a pan‐cancer network‐based approach to estimate cell line drug sensitivity; however, these models uncovered biological correlates of response rather than offering causally quantitative predictions. In other words, their results suggest plausible biological processes associated with drug responses, rather than a quantitative relationship between these.^[^ ^24^ ^]^

The integration of AI‐based methods into population PK and PD modeling has enhanced covariate screening and improved predictive modeling of variability in drug responses across populations—but still relies on pattern recognition rather than defining mechanistic, quantitative predictions.^[^ ^25^ ^]^ For example, a review about the use of AI‐based methods in modeling disease trajectories and treatment outcomes in clinical settings emphasized that these techniques help reveal predictors of efficacy and safety—supporting personalized insights—yet again focusing on associations, not deterministic quantification.^[^ ^26^ ^]^

The frequent but inaccurate attempt to identify relationships in systems that should be better assessed or quantified as associations led to false expectations or miss‐data interpretations. In CADD or CABioCD, a common (but not accurate) practice is trying to establish quantitative relationships between docking scores and biological measurements such as IC_50_s or other in vivo‐derived measurements. Similarly, other computer‐derived scores to prioritize compounds in virtual screenings (e.g., similarity values, predicted affinity, or scores obtained from data fusion or a combination of two or more methodologies) are, at best, ‘associated’ but not necessarily ‘related’ with biological activity, especially if the activity is measured in a complex environment.

**Figure 1:**
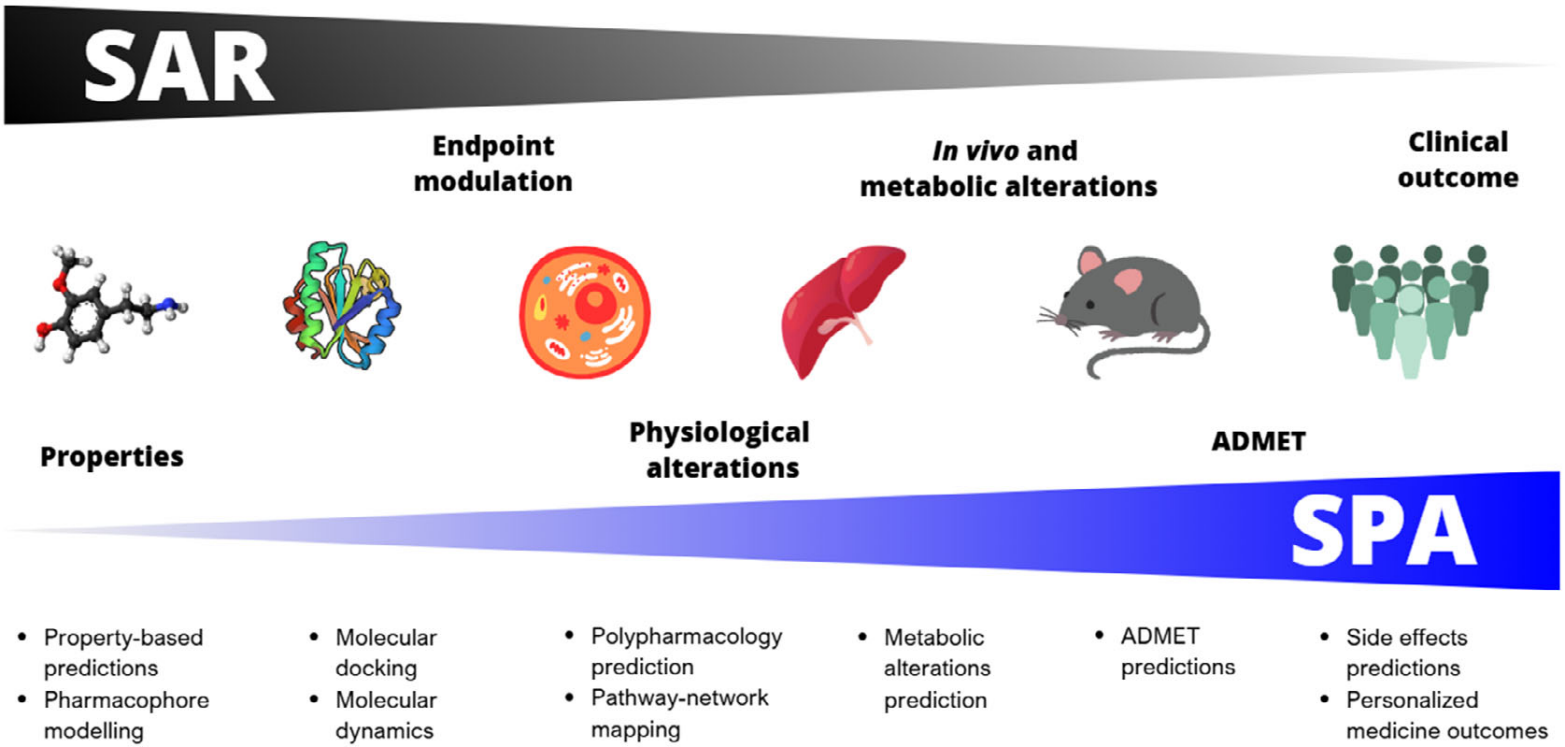
Boundaries and intersection of SAR and SPA. SARs establish a direct relationship between calculated properties (e.g., biological activity) and their experimental value. SPAs establish an indirect relationship between calculated properties and observed experimental phenomena. Physicochemical phenomena tend to be modeled using methods compatible with SAR methods, while chemical‐biological or clinical phenomena tend to be modeled using methods compatible with SPA methods.

Although conceptually distinct, there is often a thin line between SARs and SPAs. This boundary frequently blurs due to the inherent complexity and heterogeneity of the data analyzed. In practice, it is not always straightforward to delineate where one concept ends and the other begins (**Figure** **1**), underscoring the importance of explicitly defining research objectives when applying in silico methodologies. For instance, predictive studies based on physicochemical properties, pharmacophore modeling, or molecular docking should not be expected to fully explain in vitro, in vivo, or clinical phenomena. In such cases, it would be more appropriate to conduct SPAs between silico studies that rely on direct molecular interactions with more complex phenomena that are linked (e.g., associated) but do not depend entirely on those interactions. Likewise, predictions of ADMET profiles or side effects in specific populations, while valuable, should not be uncritically generalized into universal metrics or concepts. Clarifying whether a study seeks to establish SARs or SPAs is therefore essential to avoid methodological overreach and bias, and to guarantee that computational findings are interpreted within the appropriate biological and translational context. In general, as outlined in Figure 1, linking chemical structures with complex and multifactorial outcomes (e.g., in vivo and metabolic alterations, ADMET results, and clinical outcomes—see Table 2 for specific examples) should be better studied under the SPA concept. In contrast, traditional SAR methods should be used to characterize the link between chemical structures and phenomena that depend more directly on the chemical structure itself (examples are summarized in Table 1).

### 4.1 Quantitative Assessment of SPAs

The quantitative evaluation of SPAs has increasingly relied on statistical and machine learning frameworks capable of handling high‐dimensional and heterogeneous datasets. Classical regression and multivariate analysis remain useful, yet more advanced approaches such as Bayesian inference provide a principled way to integrate prior knowledge, quantify uncertainty, and improve predictive power.^[^ ^27^ ^,^ ^28^ ^]^ Recent applications demonstrate how Bayesian hierarchical models, random forests, and deep learning architectures can be used to uncover nonlinear relationships between chemical features and biological or toxicological endpoints, as seen in predictive toxicology and drug safety assessment.^[^ ^29^ ^,^ ^30^ ^]^ Moreover, contemporary studies have extended these statistical evaluations to large chemical libraries, where SPAs are tested across thousands of compounds to improve robustness and generalizability of models.^[^ ^31^ ^,^ ^32^ ^]^

Quantitative assessments of SPAs have also advanced through diverse statistical learning strategies. For instance, generalized linear models and mixed‐effects models enable the incorporation of covariates and account for repeated measures across experimental settings, while dimensionality‐reduction techniques such as principal component analysis or partial least squares facilitate the exploration of high‐dimensional chemical descriptors in relation to biological outcomes.^[^ ^28^ ^]^ Ensemble learning methods—including random forests and gradient boosting—offer powerful nonparametric alternatives that capture complex, nonlinear associations and have been applied to predictive toxicology and virtual screening tasks.^[^ ^29^ ^]^ Moreover, the integration of deep learning models provides opportunities to couple chemical representations with clinical or multiomics datasets, thereby expanding the scope of SPA analysis from single endpoints to system‐level predictions.^[^ ^30^ ^]^ Collectively, these approaches offer flexible, data‐driven perspectives that can uncover both global patterns and fine‐grained associations within complex datasets.

Parallel to molecular SPAs, there are numerous examples of indirect effects in environmental contexts, where the observed outcomes are linked not to structural variations but to differences in concentration, duration of exposure, or ecological setting. For instance, the association of perfluorooctanoic acid exposures with the increase in cancer incidence^[^ ^33^ ^]^ through contaminated water. Such associations cannot be classified as classical SPAs, since they do not reflect structural diversity, but rather dose–response or exposure–outcome association with important epidemiological implications. Similarly, the association between poly‐fluoroalkyl substances (PFAS) and cancer incidence^[^ ^34^ ^]^ was decoded by applying spatial and Bayesian methods to link historical PFAS exposures in drinking water to regional cancer incidence, accounting for residential mobility, coexposures, and contextual factors. These examples highlight the importance of distinguishing molecular‐level SARs/SPAs from broader epidemiological or environmental associations. While both domains employ quantitative models, the scope of inference differs, and avoiding overgeneralization is crucial when translating exposure‐based findings into risk assessment or policy decisions.

## 5 Discussion

In EDA, researchers typically search for patterns within data that may be broadly categorized as either relationships or associations, depending on the nature of the underlying phenomena. These patterns serve as the groundwork for deeper understanding, and in complex systems, often require simplification through reductionist approaches. This simplification facilitates the identification of underlying associations or relationships, which may be either qualitative or quantitative in nature.

Distinguishing between associations and relationships is more than a semantic nuance; it significantly influences how data is interpreted and how information is transformed into knowledge. This distinction can ultimately lead to the development of more accurate and validated models. Misinterpreting associations as true relationships—or vice versa—can result in flawed conclusions, especially within in silico analyses.

In this context, an important question emerges: do AI‐based methods primarily uncover relationships, identity associations, or both? This question merits detailed exploration, particularly through case studies that illustrate how AI‐based models detect either statistical associations or mechanistic relationships—and the consequences of misinterpreting one for the other.

The concept of ‘bioactivity disruptions’ exemplifies how associations are leveraged in the drug discovery and development pipeline. These disruptions represent valuable associative patterns that provide insight into the multifactorial nature of biological systems and drug responses.^[^ ^35^ ^]^

In highly complex systems such as clinical data and large‐scale clinical studies, the focus often shifts from relationships to associations. For example, when investigating links between chemical entities and clinical outcomes, researchers typically seek statistically significant associations rather than causal relationships, given the complexity and variability of human biology.

Although this manuscript centers on drug discovery and development, the underlying principles—particularly those related to the SPA—can be extended to the biologically relevant chemical space (BioReCS) at a broader scale. As recently discussed in the literature, BioReCS encompasses compounds of biological interest across multiple domains beyond pharmaceuticals.^[^ ^36^ ^]^ These include agrochemistry, natural products, sensory science, and food chemistry. Moreover, BioReCS captures structurally or functionally reactive molecules, including promiscuous, poly‐active compounds, and those with side effects such as toxicity or allergenicity.

## 6 Conclusions

In the context of drug discovery and development, herein we propose that the concept of SPA offers a broader and more generalizable framework than the traditional SPR approach. SPA allows a more flexible and realistic exploration of the complex connections between chemical structures and biological responses, particularly when true or causal relationships are difficult to establish. In practical medicinal chemistry and CADD, or more broadly, CABioCD, researchers frequently focus on identifying associations rather than definitive relationships. This shift arises from the recognition that biological responses are influenced by multifactorial and contextual elements, such as assay conditions and environmental parameters (e.g., pH, or oxidation levels), which extend far beyond chemical structure alone. A compelling illustration of the lack of direct chemical structure–biological response relationships can be seen in clinical settings, where biological responses are highly complex, variable, and often person‐dependent. In such cases, identifying associations is often a more scientifically accurate and productive approach than seeking rigid structure–activity or structure‐property relationships.

The misuse of the SAR concept can lead to misconceptions and overinterpretations, particularly concerning the predictive power and scope of CADD or CABioCD methodologies. In contrast, the notion of associations implies a more flexible, yet often more realistic, link between phenomena, helping to set appropriate expectations in data‐driven analyses.

This manuscript brings together insights from both medicinal and computational chemists to advocate for a shift in the interpretation of structural and biological data. In analogy with SAR studies, SPA analysis has wide‐reaching implications across medicinal chemistry and the broader drug discovery process. Its applicability extends naturally to the BioReCS, a framework that encompasses active, inactive, promiscuous, and reactive molecules of interest across multiple domains.

Given that the concept of chemical space plays a pivotal role in SAR explorations and in distinguishing active versus nonactive regions, a natural perspective lies in the formal integration of SPA within this concept. We propose the concept of Structure Property Associations in Chemical SpacE (SPACE) as an extension of exploratory SPA analysis, bringing new insights into how associations manifest and propagate across chemical space.

## Conflict of Interest

The authors declare no conflict of interest.

## Author Contributions

**Edgar** **López‐López**: formal analysis (equal); investigation (equal); writing—original draft (lead); writing—review and editing (equal). **J. Israel Espinoza‐Castañeda**: formal analysis (equal); investigation (equal); writing original draft (equal); writing—review and editing (equal). **Karina Martinez‐Mayorga**: conceptualization (lead); formal analysis (equal); funding acquisition (lead); investigation (equal); writing—original draft (equal); writing—review and editing (lead). **José L. Medina‐Franco**: conceptualization (lead); formal analysis (equal); investigation (equal); writing—original draft (lead); writing—review and editing (equal).
